# Virtual EMO-Mind: Exploring the Benefits of Virtual Mindfulness for Heart Rate Variability and Emotional Skills in Young Students

**DOI:** 10.1007/s10484-025-09759-1

**Published:** 2026-01-03

**Authors:** Amaiur Olarza, Goretti Soroa, Aitor Aritzeta, Rosa Mindeguia

**Affiliations:** 1 Department of Basic Psychological Process and Development, Faculty of Psychology, University of the Basque Country (UPV/EHU), Tolosa Avenue, 70, 20018 San Sebastian, Spain; 2 Department of Clinical and Health Psychology and Research Methodology, Faculty of Psychology, University of the Basque Country (UPV/EHU), San Sebastian, Spain; 3 Departament of Business Organization, Faculty of Economics and Business, University of the Basque Country (UPV/EHU), San Sebastian, Spain

**Keywords:** Mindfulness, Virtual reality, Children, Heart rate variability

## Abstract

Emotion regulation is essential for psychological development, and mindfulness has shown promise in enhancing emotional, cognitive, and physiological outcomes. However, traditional mindfulness practices can be challenging for children. Virtual reality offers an engaging platform to improve adherence, but its impact on children remains underexplored. This study aimed to assess the effectiveness of a short virtual reality-based mindfulness programme in improving heart rate variability and emotional skills in primary school children. The study involved 127 children aged 9–12 years (Years 5 and 6). Heart rate variability, a biomarker of autonomic nervous system activity, was measured across four virtual reality mindfulness sessions, while emotional skills were assessed pre- and post-programme. Results showed significant heart rate variability improvements, with increased high frequency power and physiological coherence indicating enhanced parasympathetic activity, relaxation, and emotional regulation. Although low frequency power decreased significantly after the first session, its interpretation as a marker of sympathetic activity remains controversial, warranting cautious analysis. In addition, the intervention improved emotional skills, particularly emotional clarity and the ability to repair emotions. Younger children (Year 5) showed greater heart rate variability gains than older peers, likely due to higher emotional plasticity during this developmental stage. These findings highlight the potential of virtual reality-based mindfulness to enhance heart rate variability, supporting better physiological and emotional regulation in children. Future research should focus on the long-term effects of these interventions and their broader applicability to promote well-being and resilience in educational and therapeutic settings.

## Introduction

### Emotion Regulation in Childhood

Emotion regulation in childhood is a fundamental aspect of psychological development and well-being (Sanchis-Sanchis et al., 2020). However, many children find it difficult to manage their emotions effectively, leading to behavioural problems, academic difficulties and problematic interpersonal relationships (Datu & King, 2018; Goldin et al., 2019). Additionally, stress experienced during childhood can further complicate the development of good emotional skills (Sciaraffa et al., 2018). It is therefore essential to develop accessible and effective methods for improving emotion regulation in children. In this context, mindfulness interventions may be of interest, as they have been found to have cognitive, social, emotional and physiological benefits (Weare, 2019), including improvements in heart rate variability (HRV), a key indicator of emotion regulation and good balance in the autonomic nervous system (ANS) (Christodoulou et al., 2020).

### Mindfulness-Based Interventions (MBIs)

Mindfulness involves conscious, non-judgmental focus on the present moment (Baer & Krietemeyer, 2006; Kabat-Zinn, 1994), promoting full awareness of current thoughts, emotions and sensations (Levit-Binnun et al., 2021). In recent years, MBIs have attracted considerable attention due to their potential to reduce stress, anxiety, depression and other psychological disorders (Shapero et al., 2018). These interventions, which focus on present-moment experiences, have demonstrated efficacy in reducing symptom severity comparable to cognitive-behavioural therapy (Bhattacharya & Hofmann, 2023).

MBIs have had promising results in terms of improving cognitive and socio-emotional outcomes in children and adolescents (Zenner et al., 2014). Meta-analyses have found significant positive effects of MBIs on executive functioning, attention, depression, anxiety/stress and maladaptive behaviours in comparison with control groups (Dunning et al., 2019). Specifically for preadolescents, MBIs have demonstrated benefits in relation to attention, behaviour regulation, positive emotions, self-evaluation and social competence (Kander et al., 2024). Additionally, a randomised controlled trial of an MBI programme for elementary school students observed improvements in cognitive control, stress physiology, empathy, perspective-taking and prosocial behaviour (Schonert-Reichl et al., 2015).

MBIs can also effectively improve emotion regulation skills, especially among children in need of additional support (Rowland et al., 2023) or those with self-regulation difficulties (Bockmann & Yu, 2023). Similarly, Pickerell et al. (2023) found that MBIs and cognitive-behavioural interventions at school can enhance emotion regulation among 7-to-12-year-old children, improving emotional awareness and increasing positive emotions.

Although mindfulness-based interventions (MBIs) have shown general benefits in children, their effectiveness may vary depending on individual characteristics such as age and gender. Regarding age, studies have suggested that MBIs tend to be more effective among older children and adolescents, who are, developmentally speaking, cognitively better equipped (i.e., more brain executive functions) to engage in mindfulness practices (López-Alarcón et al., 2020; Xie et al., 2021). Similarly, gender differences have been widely reported. Girls often show greater benefits from MBIs than boys, particularly in terms of emotional regulation and health-related quality of life (Gálvez-Tan & Alampay, 2022). This may be due to greater engagement, increased emotional openness, or higher gains in self-compassion among girls (Bluth et al., 2017; Kang et al., 2018). Moreover, the long-term effects of MBIs, such as continued improvements after several weeks, appear more sustained among girls and older students (Lassander et al., 2021).

### Virtual Reality Mindfulness-Based Interventions (VRMBIs)

Practicing mindfulness, however, requires intentional effort and can be challenging, especially for children (Benchimol-Elkaim et al., 2024; Kelly et al., 2022). Over the past decade, psychological therapies have increasingly made use of the support offered by Information and Communication Technology (ICT). In this field, virtual reality (VR) stands out for its ability to enhance mindfulness (Arpaia et al., 2022). VR refers to computer programmes that provide immersive 3D visual experiences, allowing users to interact with environments that feel real and stimulating multiple senses to create an immersive experience (Lopreiato, 2016). This technology has proven useful in increasing adherence to therapeutic interventions, such as those related to mindfulness practice and other psychological therapies (Rose et al., 2018).

VRMBIs have been more extensively researched in adults, showing significant benefits for stress management, well-being (Mitsea et al., 2022), mood (Ma et al., 2023), regulation of negative emotions (Kwon et al., 2020) and depression (Navarro-Haro et al., 2019). Nevertheless, recent studies have explored the potential of VR interventions for improving emotion regulation in children and adolescents. Positive results have been found for VR-based programmes in terms of enhancing emotional expression, emotion regulation and social interaction skills (Yuan & Ip, 2018), Carballo-Marquez et al. (2024) explored a preventive VR paradigm in a school setting, designed to improve emotion regulation and reduce symptoms of depression and anxiety in children and adolescents. The feasibility of VR for emotion regulation training in youths aged 12–15 years has also been studied, with the results suggesting that VR can effectively evoke strong emotional reactions, allowing users to practice emotion regulation in controlled environments (Kitson, 2022).

Although the use of VR in childhood is gradually becoming more widespread, research on the fusion of VR and mindfulness remains limited. Some studies, however, suggest that VRMBIs may indeed be effective for use with children. For example, VRMBIs have been observed to reduce anxiety in paediatric surgical patients (Benchimol-Elkaim et al., 2024), and a pilot study demonstrated the feasibility and acceptability of a VR-based mindfulness intervention for children with inflammatory bowel disease, reporting improvements in anxiety and pain (Wren et al., 2021).

### Heart Rate Variability (HRV)

Mindfulness has been associated with various physiological changes that affect both the nervous system and hormone production. From a neurobiological perspective, regular mindfulness practice has been linked to structural modifications in brain regions such as the prefrontal area, amygdala, and hippocampus. These structural changes correlate with improvements in attention, cognitive flexibility, thought regulation, emotion regulation and anxiety (González-Palau & Medrano, 2022). At the hormonal level, mindfulness practice has been associated with reduced levels of the stress hormone cortisol, suggesting an improvement in the regulation of the hypothalamic-pituitary-adrenal axis (Carro et al., 2023).

HRV refers to variations in the time intervals between heartbeats (RR intervals) and is considered an indicator of vagus nerve activity, which is part of the parasympathetic nervous system (PNS). HRV is associated with the flexibility and adaptability of the ANS (Shaffer & Ginsberg, 2017). Higher HRV suggests a predominance of the PNS, indicating a greater capacity for controlling emotions, thoughts, and behaviors. Higher HRV has also been linked to better psychosocial adjustment among children (Eisenberg et al., 2010). In contrast, lower HRV values indicate a disruption in homeostatic processes, potentially due to factors like chronic stress and psychopathology (McCraty & Shaffer, 2015). Reduced HRV values are associated with negative affective states (Gullett et al., 2023) and emotional instability (Blahunkova et al., 2019). HRV can therefore be used as a biomarker for adaptive social behavior, stress resilience, and self-control skills (Perna et al., 2020).

Porges’ polyvagal theory (1995) suggests a relationship between the vagus nerve and emotions, linking emotion regulation to HRV. According to this theory, the vagal nervous system has a hierarchical structure composed of three functional subsystems that activate, in sequential order (Porges, 1997, 2001): (1) the ventral vagal complex, (2) the sympathoadrenal system, and (3) the dorsal vagal complex. A reduction in heart rate and higher HRV suggest greater activity of the ventral vagal system, promoting a state of calm and protection and inhibiting the fight-or-flight reactions of the sympathetic nervous system (SNS).

HRV can be evaluated using time-domain and frequency-domain analyses (Urbanik et al., 2018). In the time domain, common measures include SDNN (standard deviation of normal-to-normal (NN) intervals), RMSSD (root mean square of successive differences between NN intervals), and pNN50 (percentage of successive NN intervals that differ by more than 50 milliseconds). While SDNN reflects total variability, RMSSD and pNN50 assess short-term variability. In the frequency domain, power spectral density (PSD) is calculated using the Fast Fourier Transform (FFT). The main parameters include high frequency (HF; 0.15–0.4 Hz), Low Frequency (LF; 0.04–0.15 Hz), very low frequency (VLF; 0.003–0.04 Hz), the LF/HF ratio, and total power (TP), which encompasses all spectral components. HF is an indicator of parasympathetic activity and vagal modulation (Thayer & Lane, 2007). In contrast, LF has traditionally been viewed as reflecting both sympathetic and parasympathetic influences (Rimoldi et al., 1990; Habek et al., 2016), and has even been interpreted by some authors as a pure marker of sympathetic tone (Triggiani et al., 2016). However, this interpretation has been increasingly questioned. As early as the 1990s, Eckberg (1997) highlighted substantial evidence undermining the use of LF power as a valid index of sympathetic activity. More recent studies employing direct measurements of sympathetic nerve activity—such as muscle sympathetic nerve activity (MSNA) and positron emission tomography (PET)—have failed to establish consistent associations between LF power, the LF/HF ratio, and actual sympathetic outflow (Floras et al., 2001; Moak et al., 2007; Rahman et al., 2011). Furthermore, the LF/HF ratio, originally proposed as an index of sympathovagal balance, has been criticised due to the complex, non-linear, and non-reciprocal interactions between the sympathetic and parasympathetic branches of the autonomic nervous system (Thomas et al., 2019; Laborde et al., 2017). As a result, interpretations based on LF and LF/HF should be approached with caution, given their limited validity as reliable indicators of autonomic balance.

Recent studies have demonstrated that mindfulness therapies can positively impact HRV, triggering an increase in the body’s ability to cope with and adapt to emotional and environmental situations. Research has shown that mindfulness enhances HRV, especially in time-domain measures such as SDNN and RMSSD, and frequency-domain measures such as HF, suggesting improvements in parasympathetic function and well-being (Tung & Hsieh, 2019). In a randomised experimental study, participating in an online mindfulness practice for 10 days was found to increase both daytime and night-time HRV, indicating potential improvements in sleep (Kirk & Axelsen, 2020). Another study found that higher levels of mindfulness were associated with greater similarity in cross-pair HRV parameters, suggesting improved regulation of ANS homeostasis (Sun et al., 2019). Additionally, studies with healthcare professionals have shown that the interaction between HRV and mindfulness helps reduce emotional fatigue and increase relaxation in the workplace (Schmid & Thomas, 2021).

However, studies focused on HRV and mindfulness in children are sparse and report mixed results. In children with attention-deficit and hyperactivity disorder (ADHD), a single session of mindfulness-based training resulted in slight improvements in attention and a mild increase in vagally-mediated HRV (Robe & Dobrean, 2023). A study by Ivaki et al. (2021) found that a 16-week yoga and mindfulness programme among elementary school children showed a slight trend towards increased parasympathetic activity, though it was not statistically significant compared to the control group. An additional study employing HRV biofeedback training found increased activation in the amygdala and anterior cingulate cortex, alongside heightened mindfulness levels, although the results did not reach statistical significance (Sigafus, 2011).

Studies on VRMBI and HRV are also limited. Pascual et al. (2023) explored how VR-based guided meditations compared with standalone mobile applications. Although the results revealed significant improvements in self-reported anxiety scores in both groups, the VR group was found to have a more pronounced increase in HRV during mindfulness sessions.

In conclusion, several studies suggest that mindfulness techniques may benefit HRV, enhancing emotion regulation and physiological well-being. Nevertheless, most of this research has focused on adults, and there is a notable lack of studies focusing on children. Furthermore, no research has been conducted on the impact of VRMBIs on children. Consequently, the aim of the present study was to analyse the effectiveness of a short programme based on virtual mindfulness for improving HRV and emotional skills among primary school children. Based on previous research, we expected HRV levels to increase as participants progressed through the programme sessions and emotional capacities to improve after the intervention. We were also interested in exploring whether differences in neuroanatomical development at a young age influenced the programme’s effectiveness, causing it to offer greater benefits to older children in terms of HRV. Finally, given that older girls tend to benefit more from mindfulness programmes than older boys (Gálvez-Tan & Alampay, 2022; Lassander et al., 2021), we aimed to determine whether there were any significant variations between boys and girls in terms of HRV outcomes and emotional skills after participating in the VRMBI programme.

## Method

### Participants

Participants were 127 children from the third key stage of primary education (Years 5 and 6), aged between 9 and 12 years (*M* = 10.56 years, *SD* = 0.66). Of the sample, 52% were in Year 5 and 48% were in Year 6. The gender distribution was 55% girls and 45% boys.

The study was conducted in two schools located in the Autonomous Community of the Basque Country. The sample size was determined on the basis of the feasibility of implementing the intervention in these schools, as they had previously participated in other programmes carried out by the University of the Basque Country. Students with Autism Spectrum Disorders (ASD), significant support needs, severe neurodevelopmental disorders, severe behavioural disorders, severe mood disorders or major language difficulties were excluded from the intervention.

This study was approved by the University of the Basque Country Committee for Research Involving Humans (M10-2020-318). Informed consent was obtained from students’ parents or legal guardians and care was taken to ensure that everyone understood the intervention and agreed to participate voluntarily. The necessary ethical aspects were rigorously followed, including the right to information, personal data protection, confidentiality guarantees, non-discrimination, and the option to withdraw from the study at any time.

### Procedure

An email was sent to those schools in the Autonomous Community of the Basque Country that had previously expressed an interest in the psychological interventions carried out by the University of the Basque Country. Subsequently, meetings were held with the head teachers of the schools to present a summary of the programme and provide additional details about its objectives and main features. Practical sessions were conducted to ensure that teachers understood the intervention. Once the schools agreed to participate, we obtained informed consent from all pupils’ parents or legal guardians.

A pre-evaluation session was conducted to measure participants’ emotional levels, after which the four-session intervention programme was implemented. One session was held per week, on the same day and at the same time, with groups of three students that remained unchanged throughout all four sessions to foster trust and enable consistent dynamics. The intervention took place in an educational context and the programme was conducted at the school during regular school hours. The programme was led by an external health psychologist specialising in mindfulness and VR and sessions were held in classrooms specially adapted to facilitate the mindfulness process, with a welcoming atmosphere, dim lighting and the quietest environment possible. The space was equipped with everything required for the activity, including three computers, VR headsets, audio headphones, and the *HeartMath EmWave* sensor.

Four HRV recordings were carried out, one during each of the virtual mindfulness sessions, each lasting approximately 15 min. The aim was to evaluate the influence of the immersive environment on participants’ physiological responses during the intervention. Measurements were taken while the children were wearing the VR headsets and actively engaged in the mindfulness practice. Data recording began simultaneously with the start of the virtual scene, triggered by pressing the play button, which automatically activated the *HeartMath EmWave sensor*.

Three physiological indicators were assessed during each session: high-frequency (HF) power, low-frequency (LF) power, and physiological coherence. HF and LF values were derived through spectral analysis using Fast Fourier Transform (FFT) within the EmWave software. Physiological coherence reflects the degree of synchronisation and order in the heart rhythm pattern, particularly within the resonant frequency band (~ 0.1 Hz). It is calculated using the following formula:

$$\begin{aligned} & {\text{Physiological coherence}} \\ & \;={\text{Peak power}}/({\text{Total power}} - {\text{Peak power}}) \\ \end{aligned} $$

Peak Power refers to the maximum power within the 0.04–0.26 Hz range (typically around 0.1 Hz), and Total Power is the overall power within the 0.0033–0.4 Hz band. A more stable and sine-wave-like heart rhythm will result in higher coherence scores. These scores are interpreted on a numerical scale as follows: low (< 0.5), medium (0.5–1.0), and high (> 1.0) (McCraty & Shaffer, 2015).

These parameters are used to assess autonomic nervous system activity and emotional self-regulation. In addition, children’s emotional abilities were assessed at two time points: prior to the start of the programme and following its completion, using a pre-post design with identical questionnaires administered during both assessment sessions.

Participants engaged in mindfulness practices in virtual settings designed to simulate the different seasons of the year: spring, summer, autumn, and winter. Each experience lasted fifteen minutes and represented one season, transporting participants to different destinations. Participants were immersed in environments designed to capture their attention with natural sounds, wide panoramas, and detailed features, using VR technology and an audio guide. During the spring session, participants enjoyed the tranquillity of a forest environment; during the summer session, they travelled to Egypt to marvel at impressive desert landscapes and pyramids; during the autumn session, they had the opportunity to explore China’s diverse culture and landscapes; and in the winter session, they were exposed to peaceful scenes featuring the snow-covered North Pole.

After the intervention, an evaluation session was conducted in which participants completed the same questionnaires used in the initial assessment. Once the data had been collected, the pertinent statistical analyses were performed.

### Measures

#### HeartMath EmWave Pro (Institute of HeartMath, 2012)

The *HeartMath EmWave* is a device designed to help individuals manage stress, improve emotional regulation, and optimise performance. The system comprises a non-invasive photoplethysmographic (PPG) sensor placed on the left earlobe and connected via USB to a computer running the EmWave software. The sensor detects pulse wave signals and calculates inter-beat intervals (IBIs), thereby measuring HRV.

The *EmWave* software continuously records IBIs and analyses heart rhythm patterns in real time. It uses an algorithm to assess the order and stability of these patterns, classifying the user’s physiological state into three coherence levels. Coherence is defined as a state in which the heart rhythm becomes orderly and sine-wave-like, reflecting a synchronised balance between sympathetic and parasympathetic activity in the autonomic nervous system.

The coherence score is a dynamic numerical value ranging from 0 to 100 and is classified into three levels: low (red), medium (blue), and high (green). This numerical coherence score is based on the continuous, moment-to-moment calculation of the coherence ratio (Peak power/[Total power – Peak power]). The 0–100 scale reflects the degree of physiological synchrony, with higher scores indicating more sustained and stable coherence. Although the EmWave software maps these values into colour-coded zones for visual feedback, the underlying score is continuous and allows for a fine-grained representation of the user’s psychophysiological state over time. At the end of each session, the software calculates an overall average score and reports the percentage of time spent in each coherence zone.

These levels reflect the degree of order and stability in the heart rhythm pattern, which in turn is indicative of the individual’s psychophysiological state. A predominance of time spent in the low coherence zone suggests disordered and erratic heart rhythms typically associated with stress, anxiety, or poor emotional regulation. In such cases, a high percentage of time in the red zone indicates that the individual is likely experiencing physiological stress or dysregulation during the session.

Medium coherence reflects a moderate level of autonomic balance, indicating some degree of heart–brain alignment and improved physiological organisation, though not yet optimal. High coherence, on the other hand, is characterised by smooth, sine-wave-like heart rhythms and reflects a harmonious psychophysiological state. This level is associated with positive emotional experiences, calm focus, and efficient functioning of the autonomic nervous system. Therefore, a greater percentage of time spent in the high coherence zone (green) is interpreted as an indicator of better emotional regulation and autonomic balance.

During each session, the software tracks the percentage of time the user spends in each coherence zone and calculates an overall coherence score. This final score is computed as a weighted average of the time spent in low, medium, and high coherence states, providing an integrated indicator of physiological regulation throughout the session.

The *HeartMath EmWave* system has been used in a variety of studies focusing on the development of emotional self-regulation in children (Aritzeta et al., 2022; Cruz, 2019), as well as the reduction of disruptive behaviours (Rush et al., 2017), anxiety, and stress (Aranberri-Ruiz et al., 2022). Its capacity to provide real-time feedback on psychophysiological states makes it a valuable tool in mindfulness-based interventions within both clinical and educational settings.

#### Kubios HRV Scientific

*Kubios HRV Scientific* is a specialised software tool for analysing HRV, primarily used in research and healthcare settings. This software analyses heart rate data obtained from electrocardiogram (ECG) recordings or other heart rate monitoring devices, such as the *HeartMath EmWave*. In our case, HRV data were collected using the *HeartMath EmWave* device and subsequently uploaded to *Kubios HRV* for physiological parameter analysis.

Artefacts, which are errors or noise in the data caused by patient movements, measurement errors or electrical interferences, can distort analysis results. Kubios provides automatic and manual tools for identifying and removing these artefacts, thereby ensuring that the data are as clean and precise as possible. In our analysis, we used the preprocessing pipeline provided by *Kubios HRV Scientific*, which includes automatic artefact correction and filtering based on RR interval variability. By default, the software applies a medium-level artefact correction threshold (typically 0.25 s), detecting and correcting RR intervals that deviate substantially from a moving average trend. In general, RR intervals that deviate more than ± 20% from the previous interval are flagged as outliers and are automatically corrected through interpolation. Additionally, the software applied its default detrending method, which removes slow non-stationary trends from the RR interval time series and improves the accuracy of HRV frequency domain measures.

#### Short Trait Meta-mood Scale (TMMS)

The Short Trait Meta-mood Scale (TMMS; Salovey et al., 1995) is a shortened version of the Trait Meta-mood Scale, which was developed to assess emotional metacognition, i.e., individuals’ ability to reflect on their emotions.

The TMMS focuses on three fundamental dimensions. The first, emotional attention, evaluates the extent to which people observe and think about their emotions. It includes items measuring awareness and interest in emotional experiences (‘I think my emotions and state of mind deserve to be paid attention to’). The second dimension, emotional clarity, refers to the ability to identify and understand one’s own emotions. It assesses how individuals differentiate between emotions and understand their causes and consequences (‘I can usually define my feelings’). The third dimension, emotional repair, measures respondents’ ability to regulate their emotions effectively. This component focuses on the capacity to maintain or regain a positive emotional state after a negative experience (‘Even when I feel sad, I try to think about pleasant things’). Participants respond to each item using a 5-point Likert-type scale ranging from ‘*Strongly Disagree*’ to ‘*Strongly Agree*’. In this study, we used the 12-item shortened version of the scale (Salguero et al., 2009). It is worth noting that Gorostiaga et al. (2011) translated the Short Trait Meta-mood Scale (TMMS-23) into Basque, obtaining good internal consistency (*α* Attention = 0.84, *α* Clarity = 0.80, *α* Repair = 0.82). In the present study, internal consistency values for all dimensions were also acceptable (*α *Attention = 0.77 and *ω* Attention = 0.77; *α* Clarity = 0.72 and *ω* Clarity = 0.73; *α* Repair = 0.68 and *ω* Repair = 0.68).

#### The Emotion Regulation Questionnaire for Children and Adolescents (ERQ-CA)

The Emotion Regulation Questionnaire for Children and Adolescents (ERQ-CA; Gullone & Taffe, 2012) comprises 10 items divided into two subscales that assess emotion regulation strategies: cognitive reappraisal (6 items) and suppression (4 items). Cognitive reappraisal involves reinterpreting emotional situations to reduce their negative impact by changing one’s appraisal of stressful events. For its part, suppression focuses on consciously inhibiting visible emotional expression without necessarily altering the internal experience of the emotion, and is often used to conform to social norms.

Participants respond to each item using a 5-point Likert-type scale ranging from 1 (S*trongly Disagree*) to 5 (*Strongly Agree*). The psychometric properties of the ERQ-CA indicate good internal consistency, with *α* = 0.83 for the cognitive reappraisal subscale and *α* = 0.75 for the suppression subscale. In this study, only the suppression subscale was used (‘I keep my emotions to myself’), and the internal consistency values were moderately acceptable: *α* = 0.60 and *ω* = 0.62.

### Statistical Analysis

A general linear model with repeated measures was used to examine differences in HRV across the four intervention sessions. When a significant main effect was detected, pairwise comparisons between sessions were conducted using Bonferroni-adjusted post-hoc tests to determine which specific sessions differed significantly from each other. Following this, Student’s *t-test* for related samples was used to compare participants’ emotional capacities before and after the intervention. Effect sizes for each mean comparison were calculated using Cohen’s *d*. An analysis of covariance (ANCOVA) was conducted to explore post-intervention differences between subgroups (girls vs. boys and Year 5 vs. Year 6 students), using pre-test scores as covariates. In cases where the ANCOVA revealed significant differences between groups, an additional session-by-session analysis was conducted using a mixed-design ANOVA. This allowed us to identify the specific sessions in which those group differences emerged. Simple effects and pairwise comparisons (with Bonferroni correction) were used to clarify when and where the differences occurred. The data were analysed using the IBM SPSS software package (version 28).

## Results

Table 1 presents the means and standard deviations of the indicators assessed across the four intervention sessions. The data include high-frequency heart rate power (HF Power, n.u., normalized units), low-frequency heart rate power (LF Power, n.u.), and high HRV coherence. HF and LF heart rate power were calculated through spectral analysis of HRV using the Fast Fourier Transform (FFT), a mathematical method that transforms heart rate data into frequency components. HF power was calculated within the frequency range of 0.15–0.40 Hz, and LF power within 0.04–0.15 Hz. Both values were expressed in normalised units (n.u.).

**Table 1:** Evolution of HRV values across sessions

	M (SD)	Wilks’ lambda	F	df for Hypothesis	df for error	*p* value	BF_10_	η^2^
*HF power (n.u.)*								
S1	49.45 (12.86)^a^	0.43	47.56	3.00	107.00	< 0.001	≈ 3.45 × 10^81^	0.57
S2	58.47 (13.27) ^b^							
S3	60.09 (10.69)^b^							
S4	61.38 (9.43)^b^							
*LF power (n.u.)*								
S1	50.44 (12.89)^a^	0.43	47.46	3.00	107.00	< 0.001	≈ 3.08 × 10^81^	0.57
S2	41.39 (13.89)^b^							
S3	39.76 (10.72)^b^							
S4	38.49 (9.45)^b^							
*High coherence*								
S1	9.20 (10.67)^a^	0.55	30.04	3.00	109.00	< 0.001	≈ 1.31 × 10^72^	0.45
S2	9.28 (10.12)^a^							
S3	34.04 (40.73)^b^							
S4	53.00 (49.94)^c^							

*S1* session 1, *S4* session 4

Superscript letters indicate statistically significant differences between sessions (Bonferroni−adjusted post hoc comparisons, *p* < 0.001)

The results revealed significant changes in both HF and LF Power across the intervention sessions (*Wilks’ Lambda* = 0.43; *F* (3, 107) = 47.56 and *F* (3, 107) = 47.46 respectively; *p* < 0.001; *η*^2^ = 0.571). Post hoc comparisons with Bonferroni correction showed that HF Power increased significantly from Session 1 to Sessions 2, 3 and 4 (all *p* < 0.001), while no significant differences were observed among Sessions 2, 3 and 4. Similarly, LF Power decreased significantly between Session 1 and all subsequent sessions (*p* < 0.001), with no significant differences between Sessions 2, 3 and 4. These findings suggest that the main changes in autonomic balance occurred after the first session and were maintained over time.

Regarding physiological coherence, a significant effect was also found across sessions (*Wilks’ Lambda* = 0.55; *F* (3, 109) = 30.04; *p* < 0.001; *η*^2^ = 0.452). Bonferroni-adjusted post hoc comparisons revealed no significant difference between Sessions 1 and 2, but coherence increased significantly in Session 3 compared to the first two (*p* < 0.001), and further increased in Session 4 compared to all previous sessions (*p* < 0.001). This indicates a progressive improvement in physiological coherence, particularly after the second session.

Some changes were observed in participants’ emotional capacities (Table 2). The results of the comparison between pre-test and post-test measurements revealed significant improvements in the emotional clarity dimension (*M*_*PRE*_ = 11.91, *SD*_*PRE*_ = 2.40; *M*_*POST*_ = 11.14, *SD*_*POST*_ = 2.25), with a moderate effect size (Cohen’s *d* = − 0.44). Similarly, significant improvements were also found in the emotional repair dimension, with a post-test mean of *M* = 11.84, *SD* = 2.12, compared to a pre-test mean of *M* = 11.12, *SD* = 2.31, and a moderate effect size (Cohen’s *d* = − 0.35). In contrast, emotional attention did not show a statistically significant change (*p* = 0.102, *d* = − 0.12), though the Bayes factor (*BF*_10_ = 2.78) suggested anecdotal evidence in favour of the alternative hypothesis. For suppression (ERQ-CA), the lack of a significant difference (*p* = 0.30, *d* = 0.05) was accompanied by a Bayes factor of *BF*_10_ = 1.41, indicating inconclusive evidence, with no clear support for either hypothesis.

**Table 2:** Mean scores, standard deviations, student’s *t*-statistics, *T-*values, degrees of freedom (*df*), *p *values and cohen’s *d* statistics during the pre-test and post-test phases

	Pre-test		Post-test		Student’s t (Pre–Post)				
	M	SD	M	SD	T	df	*p*	BF_10_	Cohen’s d
*TMMS*									
EA	10.45	2.44	10.73	2.29	− 1.28	103	0.102	2.78	− 0.12
EC	11.14	2.25	11.91	2.40	− 4.59	109	< 0.001	20368.55	− 0.44
ER	11.12	2.31	11.84	2.12	− 3.66	108	< 0.001	716.95	− 0.35
*ERQ-CA*									
S	10.91	2.22	10.79	4.46	0.53	106	0.30	1.41	0.05

*EA* emotional attention, *EC* emotional clarity, *ER* emotional repair, *S* suppression

We also explored possible differences in accordance with gender (Table 3) and academic year (Table 4), to determine whether or not the programme had a differential effect on girls and boys or students from different year groups. No statistically significant differences were found in terms of gender. Nevertheless, when analysing the results in accordance with the academic year, unexpected differences were observed. Year 5 pupils showed greater improvements in physiological measures of HF (*F* (1, 124) = 3.95, *p* = 0.04, *η*^2^ = 0.03) and LF (*F* (1, 124) = 3.97, *p* = 0.04, *η*^2^ = 0.03). These findings were further confirmed by the analysis of session-by-session comparisons (Table 5), which showed significant differences in Sessions 2 and 3 for both variables (*p* < 0.001 and *p* = 0.01, respectively), with Year 5 students consistently outperforming their Year 6 peers. In addition, emotional variables also showed a differential pattern. Year 5 students obtained higher post-intervention scores in emotional attention (*F* (1, 101) = 7.08, *p* < 0.01, *η*^2^ = 0.06) and emotional clarity (*F* (1, 107) = 5.38, *p* = 0.02, *η*^2^ = 0.04). No significant differences were found in high coherence, emotional regulation, or ERQ-CA scores.

**Table 3:** Mean scores, standard deviations, ANCOVA results, and estimated marginal means (LSMeans) with standard errors (SE) for girls and boys

	Girls				Boys				F	*p*	η^2^
	Pre-test (S1)	Post-test (S4)	LSMean (S4)	SE	Pre-test (S1)	Post-test (S4)	LSMean (S4)	SE			
	*M (SD)*	*M (SD)*			*M (SD)*	*M (SD)*					
*HRV*											
HF	48.35 (13.40)	61.02 (10.16)	61.55	0.96	50.54 (11.46)	61.39 (8.57)	60.51	1.07	0.53	0.47	0.00
LF	51.55 (13.42)	38.84 (10.19)	38.31	0.97	49.32 (11.50)	38.48 (8.59)	39.37	1.07	0.54	0.46	0.00
High coherence	10.23 (11.18)	59.21 (50.75)	57.46	6.35	7.96 (9.82)	47.00 (48.61)	47.86	6.81	1.06	0.31	0.01
*TMMS*											
EA	10.32 (2.43)	11.04 (2.01)	11.10	0.25	10.59 (2.52)	10.41 (2.58)	10.34	0.28	4.17	0.05	0.04
EC	11.08 (2.02)	11.67 (2.32)	11.72	0.22	11.21 (2.58)	12.19 (2.49)	12.14	0.25	1.66	0.20	0.01
ER	11.18 (2.24)	11.85 (2.26)	11.80	0.23	10.93 (2.39)	11.80 (1.94)	11.88	0.26	0.05	0.82	0.00
*ERQ-CA*											
S	11.16 (1.98)	10.69 (2.35)	10.56	0.29	10.64 (2.50)	10.96 (2.64)	11.11	0.32	1.64	0.20	0.02

*EA* emotional attention, *EC* emotional clarity, *ER* emotional repair, *S* suppression, *S1* session 1, *S4* session 4, *LSMean* least squares mean adjusted for, *SE* standard error of the adjusted mean

**Table 4:** Mean scores, standard deviations, ANCOVA results, and estimated marginal means (LSMeans) with standard errors (SE) for year 5 and year 6

	Year 5				Year 6				F	*p*	η^2^
	Pre-test (S1)	Post-test (S4)	LSMean (S4)	SE	Pre-test (S1)	Post-test (S4)	LSMean (S4)	SE			
	*M (SD)*	*M (SD)*			*M (SD)*	*M (SD)*					
*HRV*											
HF	48.16 (13.72)	62.65 (9.32)	62.70	0.99	50.94 (11.73)	59.89 (9.42)	59.87	1.02	3.95	0.04	0.03
LF	51.74 (13.75)	37.21 (9.34)	37.17	0.99	48.94 (11.76)	39.97 (9.44)	40.01	1.02	3.97	0.04	0.03
High coherence	7.84 (9.83)	51.81 (49.78)	52.66	6.19	10.05 (11.06)	52.29 (50.15)	51.31	6.62	0.02	0.88	0.00
*TMMS*											
EA	10.69 (2.60)	11.37 (2.18)	11.24	0.26	10.24 (2.29)	10.16 (2.25)	10.27	0.25	7,08	0.00	0.06
EC	10.96 (2.31)	12.15 (2.52)	12.28	0.22	11.30 (2.19)	11.68 (2.28)	11.54	0.22	5.38	0.02	0.04
ER	11.29 (2.38)	12.06 (2.33)	11.97	0.45	10.90 (2.24)	11.65 (1.92)	11.72	0.23	0.50	0.48	0.00
*ERQ-CA*											
S	11.08 (2.49)	10.82 (2.65)	10.73	0.30	10.75 (1.95)	10.75 (2.29)	10.83	0.29	0.06	0.81	0.00

*EA* emotional attention, *EC* emotional clarity, *ER* emotional repair, *S* suppression, *S1* session 1, *S4* session 4, *LSMean* least squares mean adjusted for, *SE* standard error of the adjusted mean

**Table 5:** HF and LF power comparison by session and grade

	Session	Year 5: M (SE)	Year 6: M (SE)	Difference (5–6)	*p* value
HF	S1	48.16 (1.67)	50.94 (1.80)	− 2.78	0.26
	S2	62.76 (1.63)	53.50 (1.75)	9.27	< 0.001
	S3	62.43 (1.36)	57.40 (1.46)	5.03	0.01
	S4	62.65 (1.22)	59.89 (1.31)	2.76	0.13
LF	S1	51.74 (1.68)	48.94 (1.80)	2.8	0.26
	S2	37.09 (1.63)	46.38 (1.75)	− 9.29	< 0.001
	S3	37.42 (1.36)	42.48 (1.47)	− 5.06	0.01
	S4	37.21 (1.22)	39.97 (1.32)	− 2.76	0.13

*S1* session 1, *S2* session 2, *S3* session 3, *S4* session 4

## Discussion

The purpose of this study was to analyse the effectiveness of a short virtual mindfulness programme for improving HRV and emotional capabilities in primary school children (Years 5 and 6). The results revealed significant changes in both HF and LF power, with the most notable improvements occurring after the first session and being sustained over time. Specifically, HF power increased significantly between Session 1 and the subsequent sessions, while LF power showed a significant decrease over the same period. Moreover, physiological coherence increased progressively, especially from the third session onwards.

This increase in HF power and physiological coherence is associated with greater PNS activity, indicating improved states of relaxation and physiological recovery (Rolph, 2017). These results align with existing research linking high HRV with enhanced emotion regulation (Brown et al., 2022), greater stress resilience (Jerath et al., 2023) and reduced risk of cardiovascular diseases (Wang et al., 2022). Moreover, high HRV correlates with better physical and mental performance, suggesting that the ANS is more flexible and efficient in individuals with elevated HRV (Lehrer et al., 2020).

These physiological changes are further supported by neuroimaging research showing that mindfulness training produces changes in the functional connectivity of the prefrontal cortex, improving emotion regulation and physiological balance (Gotink et al., 2016; Hölzel et al., 2013). The improvement in HRV observed in our study is consistent with the results observed in other MBIs carried out with different groups, although consensus is still lacking in relation to the childhood phase. For instance, a mindfulness-based stress reduction (MBSR) programme was found to increase parasympathetic activity and HRV in breast cancer survivors (Wang et al., 2021). Similarly, a study conducted with healthy individuals found improvements in HF after an 8-week MBSR intervention (Nijjar et al., 2014).

On the other hand, LF power was observed to decrease. However, due to the limitations in interpreting LF power as a definitive measure of sympathetic activity, further physiological markers would be needed to confirm a reduction in SNS activity. As previously noted, the interpretation of LF power as a direct indicator of SNS activity remains controversial. While some authors have suggested that LF may reflect sympathetic influence (Cira et al., 2017), current evidence is inconclusive (Thomas et al., 2019). Therefore, caution is warranted when drawing conclusions based solely on changes in LF values. Future studies should incorporate time-domain HRV measures, such as the mean RR interval, to provide a more comprehensive and accurate interpretation of autonomic changes observed across sessions. Including such variables would help clarify the respective roles of sympathetic and parasympathetic activity in response to mindfulness-based interventions.

The results regarding emotional capabilities indicated significant improvements in emotional clarity and repair, suggesting that participants were able to better identify and manage their emotions following the intervention. This finding is consistent with that reported in the extant literature regarding the effectiveness of mindfulness for improving emotion self-regulation. For example, recent studies have shown that digitally-assisted mindfulness training can effectively develop self-regulation skills, contributing to better mental health (Mitsea et al., 2023). Similarly, VR is increasingly being explored as a potentially useful tool for facilitating mindfulness practice and supporting emotional well-being (Navarro-Haro et al., 2017). Moreover, previous research has demonstrated that emotion regulation can be enhanced through simple daily practices in school settings. A study conducted with 24 fifth-grade students showed that using accessible daily techniques via HeartMath in the classroom could boost HRV and thus improve emotion regulation (Bearden et al., 2023).

Identifying and managing emotions in childhood is essential to ensuring children’s holistic development, laying the foundations for their emotional and social well-being throughout the rest of their lives. The ability to recognise and manage emotions helps children develop self-regulation skills (McClelland et al., 2015), improve their interpersonal relationships (Blair et al., 2015) and effectively cope with stress and conflict (Yunus & Chaudhary, 2023). Furthermore, adequate emotional education fosters self-esteem, empathy and resilience, all of which are crucial for academic and personal success in later stages (Estrada, 2020; Rajni & Singh, 2016). In other words, an early focus on emotional management can help prevent mental health issues and promote balanced, healthy development.

Regarding the gender variable, no significant differences were observed between boys and girls in either HRV values or emotional capabilities. It should be noted that no previous studies conducted with school-aged children have specifically sought to examine gender differences in HRV, making it difficult to compare the results found here with those reported previously in the extant literature. In terms of emotional skills, some studies suggest that mindfulness interventions may be more effective for improving certain aspects among girls than among boys. For example, Gálvez-Tan and Alampay (2022) found that mindfulness practices have a greater impact on girls in terms of reducing the symptoms of anxiety and improving emotional acceptance.

Another of the study’s aims was to observe the differential effect of the programme on students from different year groups (Years 5 and 6 of primary school). We hypothesised that older children would achieve higher scores in HRV and emotional skills. This was based on the findings reported by previous studies, which observed that older participants tended to benefit more from mindfulness therapies and interventions aimed at improving HRV. Specifically, previous studies have found that older individuals have a greater capacity to benefit effectively from mindfulness techniques (Lassander et al., 2021; Xie et al., 2021), as well as to experience more significant improvements in HRV (Paniccia et al., 2018).

However, our results revealed unexpected differences, with younger students (Year 5) achieving better results in the HF and LF than their older counterparts. These findings were further supported by session-by-session comparisons, which revealed significant differences in Sessions 2 and 3, with Year 5 students outperforming their Year 6 peers. A similar pattern was observed in the emotional domain: Year 5 pupils obtained higher post-intervention scores in emotional attention and emotional clarity. One possible explanation is that younger students may exhibit greater cognitive and emotional plasticity, which could help them engage more effectively with new intervention techniques. At this age, children are in a critical phase of their emotional and cognitive development, which may render them more sensitive to mindfulness practices and emotion regulation. Additionally, younger students, having accumulated less stress and anxiety than older ones, may be in a more open mental and emotional state, which may in turn facilitate a more positive response to HRV interventions. These findings tentatively suggest that younger students may be more receptive to HRV interventions and the emotion regulation techniques implemented in this programme. The discrepancy between our results and those reported in the literature highlights the need for further research into the moderating effect of age and how cognitive and emotional development may influence the effectiveness of these programmes.

This study has several limitations that should be carefully considered when interpreting the results. Firstly, the absence of a control group limits the internal validity of the findings, as it prevents establishing whether the observed improvements in HRV and emotional skills were directly caused by the intervention or influenced by external variables. Secondly, the sample was limited to primary school pupils aged 9–12, which restricts the generalisability of the results to other age group. Thirdly, the intervention was brief, comprising only four 15-minute sessions. While suitable for school settings, such a short duration may not be sufficient to consolidate long-term changes in physiological or emotional regulation. Some authors argue that children may require an adaptation period before fully engaging with mindfulness practices (Ferreira-Vorkapic et al., 2015), suggesting that a longer intervention could yield more robust outcomes. In addition, although a significant decrease in LF power was observed, its interpretation as a marker of SNS activity remains controversial and should be approached with caution, particularly in the absence of complementary physiological measures such as time-domain HRV indices (e.g., mean RR interval). The lack of follow-up assessments is another key limitation, as it prevents determining whether the effects observed during the programme were sustained over time. Future studies should incorporate randomised controlled designs, include long-term follow-up assessments, and consider potential moderating variables such as age and sex, which may influence the effectiveness and underlying mechanisms of mindfulness interventions during childhood.

In conclusion, the virtual mindfulness-based programme was found to have promising benefits for the HRV and emotional capabilities of children aged 9–12 years. VR can be used to facilitate mindfulness effectively and has proven beneficial in this context. However, it is important to point out that more research is needed to evaluate the effectiveness of such child-oriented interventions in educational settings. There is currently a lack of studies exploring the impact of VRMBIs on children’s HRV, a circumstance that limits our understanding of their influence on this population group. It would be useful to carry out additional VRMBI interventions using similar procedures, sample groups and methods to enhance the robustness (or challenge) of the findings presented here.

## Data Availability

Data can be obtained from the first author upon an email request.

## References

[CR1] Aranberri-Ruiz, A., Aritzeta, A., Olarza, A., Soroa, G., & Mindeguía, R. (2022). Reducción de La Ansiedad y El estrés social En La educación primaria: Una intervención de biorretroalimentación sobre La variabilidad de La frecuencia cardíaca Centrada En La respiración. *International Journal of Environmental Research and Public Health*, *19*(16), 10181. 10.3390/ijerph191610181 36011817 PMC9407856

[CR2] Aritzeta, A., Aranberri-Ruiz, A., Soroa, G., Mindeguía, R., & Olarza, A. (2022). Autorregulación emocional En educación primaria: Un programa de intervención de biorretroalimentación de La variabilidad de La frecuencia cardíaca. *International Journal of Environmental Research and Public Health*, *19*(9), 5475. 10.3390/ijerph19095475 35564869 PMC9099602

[CR3] Arpaia, P., D’Errico, G., De Paolis, L. T., Moccaldi, N., & Nuccetelli, F. (2022). Una revisión narrativa de intervenciones Basadas En mindfulness Que utilizan La Realidad virtual. *Atención Plena*, *13*, 556–571. 10.1007/s12671-021-01783-6

[CR4] Baer, R. A., & Krietemeyer, J. (2006). Overview of mindfulness-and acceptance-based treatment approaches. In *Mindfulness-based treatment approaches: Clinician’s guide to evidence base and applications* (pp. 3–27).

[CR5] Bearden, A. G., van Oostrom, S., & Brown, S. B. (2023). The effects of heartmath heart lock-in on elementary students’ HRV and self‐reported emotion regulation skills. *Psychology in the Schools*, *60*(12), 5245–5263. 10.1002/pits.23025

[CR6] Benchimol-Elkaim, B., Khoury, B., & Tsimicalis, A. (2024). Nature-based mindfulness programs using virtual reality to reduce pediatric perioperative anxiety: A narrative review. *Frontiers in Pediatrics*, *12*, 1334221. 10.3389/fped.2024.1334221 38283632 PMC10820709

[CR7] Bhattacharya, S., & Hofmann, S. G. (2023). Mindfulness-based interventions for anxiety and depression. *Clinics in Integrated Care*, *16*, 100138. 10.1016/j.intcar.2023.100138

[CR8] Blahunkova, S., Solarikova, P., Brezina, I., Turonova, D., & Rajcani, J. (2019). Aktivita autonómneho nervového systému v kontexte osobnostného modelu big five. *Psychologie a její Kontexty*, *10*(2), 35–43. 10.15452/PsyX.2019.10.0011

[CR9] Blair, B. L., Perry, N. B., O’Brien, M., Calkins, S. D., Keane, S. P., & Shanahan, L. (2015). Identifying developmental cascades among differentiated dimensions of social competence and emotion regulation. *Developmental Psychology*, *51*(8), 1062. 10.1037/a0039472 26147773 PMC4520405

[CR10] Bluth, K., Roberson, P. N. E., & Girdler, S. S. (2017). Adolescent sex differences in response to a mindfulness intervention: A call for research. *Journal of Child and Family Studies*, *26*(7), 1900–1914. 10.1007/s10826-017-0696-6 29051700 PMC5642304

[CR11] Bockmann, J. O., & Yu, S. Y. (2023). Using mindfulness-based interventions to support self-regulation in young children: A review of the literature. *Early Childhood Education Journal*, *51*(4), 693–703. 10.1007/s10643-022-01333-2 35340825 PMC8936381

[CR12] Brown, R. L., Chen, M. A., Paoletti, J., Dicker, E. E., Wu-Chung, E. L., LeRoy, A. S., Majd, M., Suchting, R., Thayer, J., & Fagundes, C. P. (2022). Emotion regulation, parasympathetic function, and psychological well-being. *Frontiers in Psychology*, *13*, 879166. 10.3389/fpsyg.2022.879166 35992409 PMC9381823

[CR13] Carballo-Marquez, A., Garcia-Casanovas, A., Ampatzoglou, A., Rojas-Rincon, J., Fernandez-Capo, M., Gamiz-Sanfeliu, M., Garolera-Freixa, M., & Porras-Garcia, B. O. (2024). A preventive school-based paradigm using virtual reality technologies for improving emotional regulation, depressive and anxiety symptoms in children and adolescents (e-Emotio project): A randomized controlled pilot trial. *MedRxiv*. 10.1101/2024.04.17.24305984

[CR14] Carro, N., Ibar, C., D’Adamo, P., Gonzalez, D., Berg, G., Fabre, B., & Lozada, M. (2023). Hair cortisol reduction and social integration enhancement after a mindfulness-based intervention in children. *Child: Care Health and Development*, *49*(1), 73–79. 10.1111/cch.13008 35312189

[CR15] Christodoulou, G., Salami, N., & Black, D. S. (2020). The utility of heart rate variability in mindfulness research. *Mindfulness*, *11*, 554–570. 10.1007/s12671-019-01296-3

[CR16] Cira, E. K., Syed, E. H., Sánchez-Hechavarría, M. E., & Hernández-Cáceres, J. L. (2017). El análisis de La variabilidad de frecuencia cardiaca Como Una Herramienta Para evaluar Los efectos de La meditación Chi sobre La regulación cardiovascular. *Revista Cubana De Informática Médica*, *9*(1), 30–43.

[CR17] Cruz, A. (2019). *Biofeedback as an intervention to increase self-regulation in school-aged children in an urban charter school* (Doctoral dissertation, Widener University). Widener University.

[CR18] Datu, J. A. D., & King, R. B. (2018). Subjective well-being is reciprocally associated with academic engagement: A two-wave longitudinal study. *Journal of School Psychology*, *69*, 100–110. 10.1016/j.jsp.2018.05.007 30558746

[CR19] Dunning, D. L., Griffiths, K., Kuyken, W., Crane, C., Foulkes, L., Parker, J., & Dalgleish, T. (2019). Research review: The effects of mindfulness-based interventions on cognition and mental health in children and adolescents–a meta‐analysis of randomized controlled trials. *Journal of Child Psychology and Psychiatry*, *60*(3), 244–258. 10.1111/jcpp.12980 30345511 PMC6546608

[CR20] Eckberg, D. L. (1997). Sympathovagal balance: A critical appraisal. *Circulation*, *96*(9), 3224–3232. 10.1161/01.CIR.96.9.3224 9386196

[CR21] Eisenberg, N., Spinrad, T. L., & Eggum, N. D. (2010). Emotion-related self-regulation and its relation to children’s maladjustment. *Annual Review of Clinical Psychology*, *6*(1), 495–525. 10.1146/annurev.clinpsy.121208.131208 PMC3018741 20192797

[CR22] Estrada, E. (2020). Inteligencia emocional y resiliencia En adolescentes de Una institución educativa pública de Puerto Maldonado. *Ciencia Y Desarrollo*, *23*(3), 27–35. 10.21503/cyd.v23i3.2139

[CR23] Ferreira-Vorkapic, C., Feitoza, J. M., Marchioro, M., Simões, J., Kozasa, E., & Telles, S. (2015). Are there benefits from teaching yoga at schools? A systematic review of randomized control trials of yoga-based interventions. *Evidence‐Based Complementary and Alternative Medicine*, *2015*(1), 345835. 10.1155/2015/345835 26491461 PMC4600929

[CR24] Floras, J. S., Velez-Roa, S., & Edwards, D. G. (2001). Arterial baroreflex function in patients with chronic heart failure. *American Journal of Physiology-Regulatory Integrative and Comparative Physiology*, *281*(2), R468–R476. 10.1152/ajpregu.2001.281.2.R468 11448849

[CR25] Gálvez-Tan, L. J. T., & Alampay, L. P. (2022). Exploring moderators of intervention effects of a mindfulness program for Filipino children. *International Journal of School and Educational Psychology*, *10*(3), 368–382. 10.1080/21683603.2020.1856741

[CR26] Goldin, P. R., Moodie, C. A., & Gross, J. J. (2019). Acceptance versus reappraisal: Behavioral, autonomic, and neural effects. *Cognitive Affective and Behavioral Neuroscience*, *19*, 927–944. 10.3758/s13415-019-00690-7 30656602

[CR27] González-Palau, F., & Medrano, L. A. (2022). A mini-review of work stress and mindfulness: A neuropsychological point of view. *Frontiers in Psychology*, *13*, 854204. 10.3389/fpsyg.2022.854204 35496192 PMC9051328

[CR28] Gorostiaga, A., Balluerka, N., Aritzeta, A., Haranburu, M., & Alonso, I. (2011). Measuring perceived emotional intelligence in adolescent population: Validation of the short trait meta-mood scale (TMMS-23). *International Journal of Clinical and Health Psychology*, *11*(3), 523–537.

[CR29] Gotink, R. A., Meijboom, R., Vernooij, M. W., Smits, M., & Hunink, M. M. (2016). 8-week mindfulness based stress reduction induces brain changes similar to traditional long-term meditation practice—A systematic review. *Brain and Cognition*, *108*, 32–41. 10.1016/j.bandc.2016.07.001 27429096

[CR30] Gullett, N., Zajkowska, Z., Walsh, A., Harper, R., & Mondelli, V. (2023). Heart rate variability (HRV) as a way to understand associations between the autonomic nervous system (ANS) and affective states: A critical review of the literature. *International Journal of Psychophysiology*. 10.1016/j.ijpsycho.2023.08.001 37543289

[CR31] Gullone, E., & Taffe, J. (2012). The emotion regulation questionnaire for children and adolescents (ERQ-CA): A psychometric evaluation. *Psychological Assessment*, *24*(2), 409–417. 10.1037/a0025777 22023559

[CR32] Habek, M., Crnošija, L., Lovrić, M., Junaković, A., Krbot Skorić, M., & Adamec, I. (2016). Sympathetic cardiovascular and Sudomotor functions are frequently affected in early multiple sclerosis. *Clinical Autonomic Research*, *26*, 385–393. 10.1007/s10286-016-0370-x 27448576

[CR33] Hölzel, B. K., Hoge, E. A., Greve, D. N., Gard, T., Creswell, J. D., Brown, K. W., Feldman-Barrett, L., Schwartz, C., Vaitl, D., & Lazar, S. W. (2013). Neural mechanisms of symptom improvements in generalized anxiety disorder following mindfulness training. *NeuroImage: Clinical*, *2*, 448–458. 10.1016/j.nicl.2013.03.011 24179799 PMC3777795

[CR34] Institute of HeartMath. (2012). *EmWave Desktop©*. Boulder Creek, Estados Unidos.

[CR35] Ivaki, P., Schulz, S., Jeitler, M., Kessler, C. S., Michalsen, A., Kandil, F. I., Nitzschke, S. M., Stritter, W., Voss, A., & Seifert, G. (2021). Effects of yoga and mindfulness practices on the autonomous nervous system in primary school children: A non-randomised controlled study. *Complementary Therapies in Medicine*, *61*, 102771. 10.1016/j.ctim.2021.102771 34450257

[CR36] Jerath, R., Syam, M., & Ahmed, S. (2023). The future of stress management: Integration of smartwatches and HRV technology. *Sensors (Basel, Switzerland)*, *23*(17), 7314. 10.3390/s23177314 37687769 PMC10490434

[CR37] Kabat-Zinn, J. (1994). *Wherever you go there you are: Mindfulness meditation in everyday life*. Hachette Books.

[CR38] Kander, T. N., Lawrence, D., Fox, A., Houghton, S., & Becerra, R. (2024). Mindfulness-based interventions for preadolescent children: A comprehensive meta-analysis. *Journal of School Psychology*, *102*, 101261. 10.1016/j.jsp.2023.101261 38143094

[CR39] Kang, Y., Rahrig, H., Eichel, K., Niles, H. F., Rocha, T., Lepp, N. E., Gold, J., & Britton, W. B. (2018). Gender differences in response to a school-based mindfulness training intervention for early adolescents. *Journal of School Psychology*, *68*, 163–176. 10.1016/j.jsp.2018.03.004 29861026 PMC6174072

[CR40] Kelly, R. M., Seabrook, E. M., Foley, F., Thomas, N., Nedeljkovic, M., & Wadley, G. (2022). Design considerations for supporting mindfulness in virtual reality. *Frontiers in Virtual Reality*, *2*, 672556. 10.3389/frvir.2021.672556

[CR41] Kirk, U., & Axelsen, J. L. (2020). Heart rate variability is enhanced during mindfulness practice: A randomized controlled trial involving a 10-day online-based mindfulness intervention. *PloS One*, *15*(12), e0243488. 10.1371/journal.pone.0243488 33332403 PMC7746169

[CR42] Kitson, A. (2022). Investigating the feasibility of virtual reality for emotion regulation with youth. *ArXiv Preprint*. 10.48550/arXiv.2212.00002. arXiv:2212.00002.

[CR43] Kwon, J. H., Hong, N., Kim, K., Heo, J., Kim, J. J., & Kim, E. (2020). Feasibility of A virtual reality program in managing test anxiety: A pilot study. *Cyberpsychology Behavior and Social Networking*, *23*(10), 715–720. 10.1089/cyber.2019.0651 32678684

[CR44] Laborde, S., Mosley, E., & Thayer, J. F. (2017). Heart rate variability and cardiac vagal tone in Psychophysiological research–recommendations for experiment planning, data analysis, and data reporting. *Frontiers in Psychology*, *8*, 213. 10.3389/fpsyg.2017.00213 28265249 PMC5316555

[CR45] Lassander, M., Hintsanen, M., Suominen, S., Mullola, S., Vahlberg, T., & Volanen, S. M. (2021). Effects of school-based mindfulness intervention on health-related quality of life: Moderating effect of gender, grade, and independent practice in cluster randomized controlled trial. *Quality of Life Research*, *30*(12), 3407–3419. 10.1007/s11136-021-02868-4 34169412 PMC8602227

[CR46] Lehrer, P., Kaur, K., Sharma, A., Shah, K., Huseby, R., Bhavsar, J., & Zhang, Y. (2020). Heart rate variability biofeedback improves emotional and physical health and performance: A systematic review and meta analysis. *Applied Psychophysiology and Biofeedback*, *45*, 109–129. 10.1007/s10484-020-09466-z 32385728

[CR47] Levit-Binnun, N., Arbel, K., & Dorjee, D. (2021). The mindfulness map: A practical classification framework of mindfulness practices, associated intentions, and experiential Understandings. *Frontiers in Psychology*, *12*, 727857. 10.3389/fpsyg.2021.727857 34712178 PMC8545890

[CR48] López-Alarcón, M., Zurita-Cruz, J. N., Torres-Rodríguez, A., Bedia-Mejía, K., Pérez-Güemez, M., Jaramillo-Villanueva, L., Rendón-Macías, M. E., Fernández, J. R., & Martínez-Maroñas, P. (2020). Mindfulness affects stress, ghrelin, and BMI of obese children: A clinical trial. *Endocrine Connections*, *9*(2), 163–172. 10.1530/EC-19-0461 32045358 PMC7040861

[CR49] Lopreiato, J. O. (2016). *Healthcare simulation dictionary*. Agency for Healthcare Research and Quality.

[CR50] Ma, J., Zhao, D., Xu, N., & Yang, J. (2023). The effectiveness of immersive virtual reality (VR) based mindfulness training on improvement mental-health in adults: A narrative systematic review. *Explore*, *19*(3), 310–318. 10.1016/j.explore.2022.08.001 36002363

[CR51] McClelland, M. M., Geldhof, J., Cameron, G., C. E., & Wanless, S. B. (2015). Development and self-regulation. In *Handbook of child psychology and developmental science* (pp. 1–43). 10.1002/9781118963418.childpsy114

[CR52] McCraty, R., & Shaffer, F. (2015). Heart rate variability: New perspectives on physiological mechanisms, assessment of self-regulatory capacity, and health risk. *Global Advances in Health and Medicine*, *4*(1), 46–61. 10.7453/gahmj.2014.073 25694852 PMC4311559

[CR53] Mitsea, E., Drigas, A., & Skianis, C. (2022). Mindfulness for anxiety management and happiness: The role of VR, metacognition, and hormones. *Technium BioChemMed*, *3*(3), 37–52. 10.47577/biochemmed.v3i3.7343

[CR54] Mitsea, E., Drigas, A., & Skianis, C. (2023). Digitally assisted mindfulness in training self-regulation skills for sustainable mental health: A systematic review. *Behavioral Sciences*, *13*(12), 1008. 10.3390/bs13121008 38131865 PMC10740653

[CR55] Moak, J. P., Goldstein, D. S., Eldadah, B. A., Saleem, A., Holmes, C., Pechnik, S., & Sharabi, Y. (2007). Supine low-frequency power of heart rate variability reflects baroreflex function, not cardiac sympathetic innervation. *Heart Rhythm: the official Journal of the Heart Rhythm Society*, *4*(12), 1523–1529. 10.1016/j.hrthm.2007.07.019 PMC2204059 17997358

[CR56] Navarro-Haro, M. V., López-del-Hoyo, Y., Campos, D., Linehan, M. M., Hoffman, H. G., García-Palacios, A., Modego-Alarcón, M., Borao, L., & García-Campayo, J. (2017). Meditation experts try virtual reality mindfulness: A pilot study evaluation of the feasibility and acceptability of virtual reality to facilitate mindfulness practice in people attending a mindfulness conference. PloS ONE, 12(11), e0187777. 10.1371/journal.pone.0187777 PMC5699841 29166665

[CR57] Navarro-Haro, M. V., Modrego-Alarcón, M., Hoffman, H. G., López-Montoyo, A., Navarro-Gil, M., Montero-Marin, J., García-Palacios, A., Borao, L., & García-Campayo, J. (2019). Evaluation of A mindfulness-based intervention with and without virtual reality dialectical behavior therapy^®^ mindfulness skills training for the treatment of generalized anxiety disorder in primary care: A pilot study. *Frontiers in Psychology*, *10*, 55. 10.3389/fpsyg.2019.00055 30745888 PMC6360930

[CR58] Nijjar, P. S., Puppala, V. K., Dickinson, O., Duval, S., Duprez, D., Kreitzer, M. J., & Benditt, D. G. (2014). Modulation of the autonomic nervous system assessed through heart rate variability by a mindfulness based stress reduction program. *International Journal of Cardiology*, *177*(2), 557–559. 10.1016/j.ijcard.2014.08.116 25179555

[CR59] Paniccia, M., Verweel, L., Thomas, S., Taha, T., Keightley, M., Wilson, K. E., & Reed, N. (2018). Heart rate variability in healthy non-concussed youth athletes: Exploring the effect of age, sex, and concussion-like symptoms. *Frontiers in Neurology*, *8*, 753. 10.3389/fneur.2017.00753 29403426 PMC5778119

[CR60] Pascual, K., Fredman, A., Naum, A., Patil, C., & Sikka, N. (2023). Should mindfulness for health care workers go virtual? A mindfulness-based intervention using virtual reality and heart rate variability in the emergency department. *Workplace Health & Safety*, *71*(4), 188–194. 10.1177/21650799221123258 36377263

[CR61] Perna, G., Riva, A., Defillo, A., Sangiorgio, E., Nobile, M., & Caldirola, D. (2020). Heart rate variability: Can it serve as a marker of mental health resilience? Special section on translational and neuroscience studies in affective disorders section Editor, Maria nobile MD, phd. *Journal of Affective Disorders*, *263*, 754–761. 10.1016/j.jad.2019.10.017 31630828

[CR62] Pickerell, L. E., Pennington, K., Cartledge, C., Miller, K. A., & Curtis, F. (2023). The effectiveness of school-based mindfulness and cognitive behavioural programmes to improve emotional regulation in 7–12-year-olds: A systematic review and meta-analysis. *Mindfulness*, *14*(5), 1068–1087. 10.1007/s12671-023-02131-6

[CR63] Porges, S. W. (1995). Orienting in a defensive world: Mammalian modifications of our evolutionary heritage. A polyvagal theory. *Psychophysiology*, *32*(4), 301–318. 10.1111/j.1469-8986.1995.tb01213.x 7652107

[CR64] Porges, S. W. (1997). Emotion: An evolutionary by-product of the neural regulation of the autonomic nervous system. *Annals of the New York Academy of Sciences*, *807*, 62–77. 9071344 10.1111/j.1749-6632.1997.tb51913.x

[CR65] Porges, S. W. (2001). The polyvagal theory: Phylogenetic substrates of a social nervous system. *International Journal of Psychophysiology*, *42*(2), 123–146. 10.1016/S0167-8760(01)00162-3 11587772

[CR66] Rahman, F., Pechnik, S., Gross, D., Sewell, L., & Goldstein, D. S. (2011). Low frequency power of heart rate variability reflects baroreflex function, not cardiac sympathetic innervation. *Clinical Autonomic Research*, *21*(3), 133–141. 10.1007/s10286-010-0098-y 21279414 PMC3094491

[CR67] Rajni, M., & Singh, S. (2016). A review of emotional intelligence on self esteem: It’s impact on adolescents stage. *The International Journal of Indian Psychology*, *3*(3), 40–44. 10.25215/0303.081

[CR68] Rimoldi, O., Pierini, S., Ferrari, A., Cerutti, S., Pagani, M., & Malliani, A. (1990). Analysis of short-term oscillations of R-R and arterial pressure in conscious dogs. *American Journal of Physiology*, *258*(5), H967–H976. 2109943 10.1152/ajpheart.1990.258.4.H967

[CR69] Robe, A., & Dobrean, A. (2023). The effectiveness of a single session of mindfulness-based cognitive training on cardiac vagal control and core symptoms in children and adolescents with attention-deficit/hyperactivity disorder (ADHD): A preliminary randomized controlled trial. *European Child and Adolescent Psychiatry*, *32*(10), 1863–1872. 10.1007/s00787-022-02005-7 35608666 PMC9128328

[CR70] Rolph, G. W. (2017). Effects of mindfulness on perceived stress levels and heart rate variability. *ArXiv Preprint*. 10.48550/arXiv.1708.08006. arXiv:1708.08006.

[CR71] Rose, T., Nam, C. S., & Chen, K. B. (2018). Immersion of virtual reality for rehabilitation-Review. *Applied Ergonomics*, *69*, 153–161. 10.1016/j.apergo.2018.01.009 29477323

[CR72] Rowland, G., Hindman, E., & Hassmén, P. (2023). Do group mindfulness-based interventions improve emotion regulation in children? A systematic review. *Journal of Child and Family Studies*, *32*(5), 1294–1303. 10.1007/s10826-023-02544-w

[CR73] Rush, K. S., Golden, M., & Mortenson, B. P. (2017). Los efectos de Un programa de atención plena y biorretroalimentación sobre Las Conductas Dentro y Fuera de La Tarea de estudiantes Con Trastornos emocionales Del Comportamiento. *Contemporary School Psychology*, *21*, 347–357. 10.1007/s40688-017-0140-3

[CR74] Salguero, J. M., Berrocal, P. F., Aranda, D. R., & González, R. C. (2009). Propiedades psicométricas de la versión reducida de la Trait Meta-Mood Scale: TMMS-12. In *Avances en el estudio de la inteligencia emocional* (pp. 129–134). Fundación Marcelino Botín.

[CR75] Salovey, P., Mayer, J. D., Goldman, S. L., Turvey, C., & Palfai, T. P. (1995). Emotional attention, clarity, and repair: Exploring emotional intelligence using the trait Meta-Mood. In J. W. Pennebaker (Ed.), *Emotion, disclosure, and health* (pp. 125–151). American Psychological Association. 10.1037/10182-006

[CR76] Sanchis-Sanchis, A., Grau, M. D., Moliner, A. R., & Morales-Murillo, C. P. (2020). Effects of age and gender in emotion regulation of children and adolescents. *Frontiers in Psychology*, *11*, 946. 10.3389/fpsyg.2020.00946 32528367 PMC7265134

[CR77] Schmid, R. F., & Thomas, J. (2021). The interactive effects of heart rate variability and mindfulness on indicators of well-being in healthcare professionals’ daily working life. *International Journal of Psychophysiology*, *164*, 130–138. 10.1016/j.ijpsycho.2021.01.012 33548348

[CR78] Schonert-Reichl, K. A., Oberle, E., Lawlor, M. S., Abbott, D., Thomson, K., Oberlander, T. F., & Diamond, A. (2015). Enhancing cognitive and social–emotional development through a simple-to-administer mindfulness-based school program for elementary school children: A randomized controlled trial. *Developmental Psychology*, *51*(1), 52. 10.1037/a0038454 25546595 PMC4323355

[CR79] Sciaraffa, M. A., Zeanah, P. D., & Zeanah, C. H. (2018). Understanding and promoting resilience in the context of adverse childhood experiences. *Early Childhood Education Journal*, *46*, 343–353. 10.1007/s10643-017-0869-3

[CR80] Shaffer, F., & Ginsberg, J. P. (2017). An overview of heart rate variability metrics and norms. *Frontiers in Public Health*, *5*, 258. 10.3389/fpubh.2017.00258 29034226 PMC5624990

[CR81] Shapero, B. G., Greenberg, J., Pedrelli, P., de Jong, M., & Desbordes, G. (2018). Mindfulness-based interventions in psychiatry. *Focus*, *16*(1), 32–39. 10.1176/appi.focus.20170039 29599651 PMC5870875

[CR82] Sigafus, P. (2011). Heart rate variability biofeedback and mindfulness: A functional neuroimaging study. *Cleveland Clinic Journal of Medicine*, *78*(1), S102–S102.

[CR83] Sun, S., Hu, C., Pan, J., Liu, C., & Huang, M. (2019). Trait mindfulness is associated with the self-similarity of heart rate variability. *Frontiers in Psychology*, *10*, 314. 10.3389/fpsyg.2019.00314 30873070 PMC6403186

[CR84] Thayer, J. F., & Lane, R. D. (2007). The role of vagal function in the risk for cardiovascular disease and mortality. *Biological Psychology*, *74*(2), 224–242. 10.1016/j.biopsycho.2005.11.013 17182165

[CR85] Thomas, B. L., Claassen, N., Becker, P., & Viljoen, M. (2019). Validity of commonly used heart rate variability markers of autonomic nervous system function. *Neuropsychobiology*, *78*(1), 14–26. 10.1159/000495519 30721903

[CR86] Triggiani, A. I., Valenzano, A., Del Percio, C., Marzano, N., Soricelli, A., Petito, A., & Babiloni, C. (2016). Resting state Rolandic mu rhythms are related to activity of sympathetic component of autonomic nervous system in healthy humans. *International Journal of Psychophysiology*, *103*, 79–87. 10.1016/j.ijpsycho.2015.02.009 25660308

[CR87] Tung, Y. H., & Hsieh, J. C. (2019). The impacts of mindfulness on heart rate variability: A brief review. *International Journal of Pharma Medicine and Biological Sciences*, *8*, 132–137. 10.18178/ijpmbs.8.4.132-137

[CR88] Urbanik, D., Podgórski, M., & Mazur, G. (2018). Heart rate variability–clinical significance. *Family Medicine and Primary Care Review*, *20*, 87–90. 10.5114/fmpcr.2018.73710

[CR90] Wang, Y. C., Wang, C. C., Yao, Y. H., & Wu, W. T. (2021). Identification of a high-risk group of new-onset cardiovascular disease in occupational drivers by analyzing heart rate variability. *International Journal of Environmental Research and Public Health*, *18*(21), 11486. 10.3390/ijerph182111486 34770003 PMC8582774

[CR89] Wang, S. J., Chang, Y. C., Hu, W. Y., Chang, Y. M., & Lo, C. (2022). The comparative effect of reduced mindfulness-based stress on heart rate variability among patients with breast cancer. *International Journal of Environmental Research and Public Health*, *19*(11), 6537. 10.3390/ijerph19116537 35682121 PMC9180838

[CR91] Weare, K. (2019). Mindfulness and contemplative approaches in education. *Current Opinion in Psychology*, *28*, 321–326. 10.1016/j.copsyc.2019.06.001 31404846

[CR92] Wren, A. A., Neiman, N., Caruso, T. J., Rodriguez, S., Taylor, K., Madill, M., River, H., & Nguyen, L. (2021). Mindfulness-based virtual reality intervention for children and young adults with inflammatory bowel disease: A pilot feasibility and acceptability study. *Children*, *8*(5), 368. 10.3390/children8050368 34063034 PMC8147916

[CR93] Xie, Q. W., Dai, X., Lyu, R., & Lu, S. (2021). Effects of mindfulness-based parallel-group interventions on family functioning and child and parent mental health: A systematic review and meta-analysis. *Mindfulness*, *12*(11), 2843–2864. 10.1007/s12671-021-01728-z

[CR94] Yuan, S. N. V., & Ip, H. H. S. (2018). Using virtual reality to train emotional and social skills in children with autism spectrum disorder. *London Journal of Primary Care*, *10*(4), 110–112. 10.1080/17571472.2018.1483000 30083244 PMC6074644

[CR95] Yunus, M., & Chaudhary, P. K. (2023). The role of emotion regulation in stress management: An overview. *Journal of Clinical Research and Applied Medicine*, *3*(1), 9–12. 10.5530/jcram.3.1.3

[CR96] Zenner, C., Herrnleben-Kurz, S., & Walach, H. (2014). Mindfulness-based interventions in schools: A systematic review and meta-analysis. *Frontiers in Psychology*, *5*, 603. 10.3389/fpsyg.2014.00603 25071620 PMC4075476

